# Genetic and Phenotypic Correlations between Performance Traits with Meat Quality and Carcass Characteristics in Commercial Crossbred Pigs

**DOI:** 10.1371/journal.pone.0110105

**Published:** 2014-10-28

**Authors:** Younes Miar, Graham Plastow, Heather Bruce, Stephen Moore, Ghader Manafiazar, Robert Kemp, Patrick Charagu, Abe Huisman, Benny van Haandel, Chunyan Zhang, Robert McKay, Zhiquan Wang

**Affiliations:** 1 Livestock Gentec Centre, Department of Agricultural, Food and Nutritional Science, University of Alberta, Edmonton, Alberta, Canada; 2 Genesus Genetics, Oakville, Manitoba, Canada; 3 Hypor Inc., Regina, Saskatchewan, Canada; 4 Research and Technology Centre, Hendrix Genetics, Boxmeer, The Netherlands; 5 Centre for Animal Science, Queensland Alliance for Agriculture & Food Innovation, University of Queensland, St Lucia, Australia; 6 McKay GENSTAT Consultants Inc., Brandon, Manitoba, Canada; Huazhong Agricultural University, China

## Abstract

Genetic correlations between performance traits with meat quality and carcass traits were estimated on 6,408 commercial crossbred pigs with performance traits recorded in production systems with 2,100 of them having meat quality and carcass measurements. Significant fixed effects (company, sex and batch), covariates (birth weight, cold carcass weight, and age), random effects (additive, litter and maternal) were fitted in the statistical models. A series of pairwise bivariate analyses were implemented in ASREML to estimate heritability, phenotypic, and genetic correlations between performance traits (n = 9) with meat quality (n = 25) and carcass (n = 19) traits. The animals had a pedigree compromised of 9,439 animals over 15 generations. Performance traits had low-to-moderate heritabilities (±SE), ranged from 0.07±0.13 to 0.45±0.07 for weaning weight, and ultrasound backfat depth, respectively. Genetic correlations between performance and carcass traits were moderate to high. The results indicate that: (a) selection for birth weight may increase drip loss, lightness of *longissimus dorsi*, and *gluteus medius* muscles but may reduce fat depth; (b) selection for nursery weight can be valuable for increasing both quantity and quality traits; (c) selection for increased daily gain may increase the carcass weight and most of the primal cuts. These findings suggest that deterioration of pork quality may have occurred over many generations through the selection for less backfat thickness, and feed efficiency, but selection for growth had no adverse effects on pork quality. Low-to-moderate heritabilities for performance traits indicate that they could be improved using traditional selection or genomic selection. The estimated genetic parameters for performance, carcass and meat quality traits may be incorporated into the breeding programs that emphasize product quality in these Canadian swine populations.

## Introduction

Swine breeding programs have mainly focused on production efficiency to increase the leanness of the carcasses in previous decades. This has led to dramatic improvement in production efficiency including leanness and feed efficiency owing to relatively moderate-to-high heritabilities. However, the importance of meat and carcass quality is growing for pig breeders to meet processor’s, packer’s, and consumer’s demands for better pork quality [1]. Genetic correlations between pork quality and carcass characteristics and other economic importance traits are, however, limited. Understanding of the genetic control of pork quality traits and their correlations with growth and performance traits are needed for Canadian swine populations to implement a successful breeding program that emphasizes product quality.

Meat quality traits are low-to-moderately heritable while carcass composition traits are moderate-to-highly heritable [2]. Latorre et al. [3] stated that the relationships between meat quality traits and growth traits are contradictory. Cameron [4] showed that selection for increased leanness reduced eating quality. Furthermore, weak negative genetic correlations between performance and meat quality traits have been reported and their magnitudes depend on breed [5]. Medium weight pigs at birth had a better tenderness and water holding capacity than light weight piglets but the intramuscular fat was higher in light piglets [6]. van Wijk et al. [7] stated that average daily gain was unfavorably correlated with subprimal cuts and with most meat quality traits. Jiang et al. [8] reported different breeds in Chinese swine industry had different meat quality and carcass characteristics. Various factors may influence the variance component estimates including the end-point adjustment, population size, sampling and available pedigree [9]. Phenotypic and genetic correlations between meat and carcass quality traits have been reported in our previous publication [2]. This study is a further investigation focusing on genetic and phenotypic correlations between performance traits with pork and carcass quality traits.

The objectives of this study were 1) to estimate heritabilities for various growth, and performance traits; and 2) to estimate phenotypic and genetic correlations between performance traits with pork quality and carcass traits in commercial crossbred pigs.

## Methods

The hogs used in this study were cared for according to the Canadian Council on Animal Care [10] guidelines.

### Animals and Management

The commercial crossbred pigs used in this study were progeny from a total of 139 sires of the Duroc boars bred to 429 F1 hybrid Landrace × Large White sows. These breeds were chosen because they are representative of a large percentage of the Canadian swine industry. They were a combination of full and half sib families representing a multi-generation family structure drawn from two breeding populations (Genesus Genetics, and Hypor Inc., Canada). Pedigree information of 15 ancestral generations comprising 9,439 individuals was available [2].

### Performance Evaluation and Housing

Piglets were born over a 2-year period from 2010 to 2012. All piglets were individually tagged and weighed at birth (birth weight, **BW**), weaned at an average age of 21 days (7.5 kg), raised in a nursery for 5 to 6 weeks, and then moved to pre-grower barn for 4 weeks. During this time, both weaning weight (**WNW**) and nursery weight (NURW) were recorded. Pigs were then randomly allocated to finishing sites for 9 weeks under commercial finishing conditions with *ad libitum* access to a canola, wheat, barley, soybean diet and water [2]. Male piglets were castrated at 3 to 5 days after birth. The end body weight (**ENDW**), ultrasound backfat depth (**UFD**), ultrasound loin depth (**ULD**), and ultrasound intramuscular fat (**UIMF**) were measured at the end of finishing test with an average body weight of 115 kg. The live body weights recorded at the birth and end of finishing were used to calculate the average daily gain (**ADG**) using the following equation: ADG = (ENDW – start test weight)/Days. Feed conversion ratio (**FCR**) was calculated based on the daily feed intake recorded by electronic feeders for some animals.

### Carcass and Meat Quality Measurements

Carcass and meat quality measurements have been described previously by Miar et al. [2]. Briefly, pigs were housed overnight at the abattoir (East 40 Packers, Brandon, Manitoba, Canada) with *ad libitum* access to water. Animals were slaughtered on a Federally inspected kill floor and handling of the animals upon arrival and before slaughter. Moreover, slaughter process was adhered to Government of Canada Guidelines. The average slaughter weight and age were 124 kg and 160 days, respectively. Hot carcass weight (**HCW**), cold carcass weight (**CCW**), and the carcass length (**CLEN**) were recorded according to Miar et al. [2]. Then, the carcasses were broken into the primal cuts and the loin was further broken into the front, back, 3-rib sample, 1-inch chop, and 4-rib sample. The chop removed at 3^rd^ and 4^th^ last rib was used to determine: (a) *longissimus dorsi* muscle area (**LEA**); (b) subcutaneous backfat depth (**FD**); (c) loin depth (**LD**); (d) texture score (**TEXS**) measured on a subjective 5-point scale (1 = extremely soft and weeping; 5 = very firm and dry; a score of 3 being normal) to determine if the loin was pale, soft and exudative (PSE); (e) subjective marbling score (**CMAR**; 1 to 6, with 0 = devoid, 1 = practically devoid, 2 = trace amount of marbling, 3 = slight, 4 = small, 5 = moderate, 6 = abundant) as determined by the National Swine Improvement Federation (NSIF) marbling charts [11] as described by Miar et al. [2].

Primal cuts of loin, ham, shoulder and belly were dissected into subprimal cuts. Untrimmed side weight (**USW**) was determined as the sum of the weights of untrimmed ham, loin, shoulder, and belly. Untrimmed shoulder (**USH**), untrimmed ham (**UHAM**) were removed from the side weight. Untrimmed loin (**ULOIN**) and belly (**UBEL**) were separated from each other. Then, subprimal cuts of ham (**THAM**), loin (**TLOIN**), picnic shoulder (**PICN**), butt (**BUTT**), belly (**TBEL**) and ribs (**RIBS**) were recorded as described by Miar et al. [2].

At the slaughterhouse, meat quality measurements were taken on *longissimus dorsi* muscle of the loin. Ultimate or 24 h pH (**PHU**), drip loss (**DL**), Minolta L*, a*, and b* (**LOINL**, **LOINA**, and **LOINB**) were taken on loin as describe by Miar et al. [2]. Minolta L*, a*, and b* measurements were taken on different muscles of ham including *gluteus medius* (**HGML**, **HGMA**, and **HGMB**), *quadriceps femoris* (**HQFL**, **HQFA**, and **HQFB**), and *iliopsoas* muscles (**HILL**, **HILA**, and **HILB**).

At the Meat Science Laboratory of University of Alberta, frozen 3-Rib and 4-Rib samples of the loin of each carcass were used to record whole loin weight (**WLW**), backfat weight (**BFW**), and rib eye weight (**REAW**) as described by Miar et al. [2]. Rib eye area was used for subsequent pork quality assays. Rib eye Minolta L*, a*, and b* values (**REAL**, **REAA**, and **REAB**) were taken using a commercial color meter (CR400, Konica-Minolta, Osaka, Japan) on a D 65 light setting which mimics daylight [2]. Cooking loss (**CL**) and shear force (**SHF**) were measured as described by Miar et al. [2]. The remainder of the loin was dissected into the muscle and fat (**RTW**), bone (**BOW**) and diaphragm.

### Statistical Analyses

There were 6,408 pigs with growth and performance records with 2,100 of them having meat quality and carcass data. The significance of the fixed effects and covariates for each trait was determined using the REML procedure of ASREML 3.0 software [12]. The significance of different random terms in the model was determined by likelihood ratio test using ASREML 3.0 software [12]. The full animal model included random direct, maternal additive genetic and common environment (litter of birth) effects. Maternal genetic and common environment effects were tested separately by comparing −2 residual log likelihoods of full and reduced (excluding the random effect of interest) models having degrees of freedom equal to the number of parameters tested. The model which best fit the data was selected. Common litter effects were significant (*P<0.05*) for BW, WNW, NURW, ENDW, ADG, UFD, ULD, HCW, CCW, LEA, PH, and DL and were not significant (*P>0.05*) for most meat quality, and carcass composition traits [2]. The maternal effect was only significant (*P<0.05*) for WNW.

Genetic and phenotypic (co)variances were estimated using a pairwise bivariate animal model by ASREML 3.0 [12]. Relevant fixed and random effects for carcass and meat quality traits were described by Miar et al. [2], and for performance traits are presented in Table 1. The final animal model included linear covariates of birth weight, whole loin weight received at the Meat Science Laboratory, cold carcass weight and slaughter age. Fixed effects including company, sex, and batch (test or slaughter batch) were fitted in the final model. In addition, additive polygenic effects for all traits, random litter effect, and maternal effect for some traits were included in the final model. The model is given by:
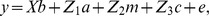
where ***y*** is the vector of phenotypic measurements, ***X*** is the incidence matrix relating the fixed effects to vector ***y***, ***b*** is the vector of fixed effects, 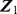 is the incidence matrix relating the phenotypic observations to the vector of polygenic (**a**) effects, 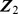 is the incidence matrix relating the phenotypic observations to the vector of maternal genetic (**m**) effects, 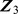 is the incidence matrix relating the phenotypic observations to the vector of common litter (**c**) effects, and **e** is the vector of random residuals.

**Table 1:** Significance of the fixed and random effects included in the models for the analysis of Performance Traits.

Traits	Fixed Effects				Random Effects		
	Company	Sex	Batch	BW^1^	Dam	Litter	Animal
**Birth weight**	**	**	**	–	NS	**	**✓**
**Weaning weight**	**	*	**	**	**	**	**✓**
**Nursery weight**	NS	**	**	**	NS	**	**✓**
**End weight**	*	**	**	–	NS	**	**✓**
**ADG**	**	**	**	–	NS	**	**✓**
**Ultrasound backfat depth**	*	**	**	–	NS	**	**✓**
**Ultrasound loin depth**	**	*	**	–	NS	**	**✓**
**Ultrasound IMF**	**	**	**	–	NS	NS	**✓**
**Feed conversion ratio**	NS	**	**	–	NS	NS	**✓**

**P<0.05; * *P<0.1*; NS: Non-significant.

1 Birth weight.

It was assumed that random effects were independent except for the covariance between the direct and the maternal additive genetic effects. In particular, the (co)variances of random variables were as follows:
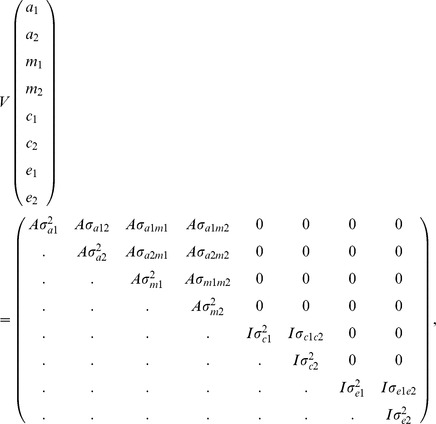



where *A* is the numerator relationship matrix, *I* is the identity matrix, 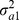, 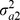, 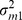, 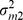, 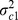, 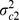, 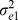 and 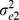 are direct additive genetic variances, maternal genetic variances, common litter effect variances and residual variance for traits 1 and 2, respectively, and 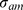 is the covariance between the direct and the maternal additive genetic effects. Variance components obtained from the bivariate analyses used to estimated heritability for each performance trait and the average estimates of corresponding pairwise bivariate analyses were reported as the heritabilities:



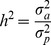



A preliminary univariate animal model for each trait was performed to obtain initial values of variance parameters that were then used in subsequent bivariate analyses. Initial values of covariance parameters were obtained by multiplying their standard deviations by their phenotypic or genetic correlations. Pairwise bivariate analyses were performed between performance traits with carcass and pork quality traits. The 2-trait individual animal model used to estimate (co)variance components, were used to calculate the phenotypic and genetic correlations as well as the heritability as implemented in ASREML 3.0 [12].

## Results and Discussion

### Means and standard deviations

Most of the performance, carcass and pork quality traits were recorded for all individuals within group. Means, standard deviations, number of measurements per trait, minimum and maximum for each performance trait are given in Table 2. The descriptive statistics for meat quality and carcass traits were previously reported by Miar et al. [2].

**Table 2:** Descriptive statistics for performance traits: number of animals per trait (n), means, SD, minimum (Min.) and maximum (Max.) values.

Traits	n	Mean	SD	Min.	Max.
**Birth weight, kg**	6408	1.53	0.35	0.50	2.90
**Weaning weight, kg**	5918	6.9	1.41	1.24	12.20
**Nursery weight, kg**	2262	37.58	7.70	10.00	60.00
**End weight, kg**	5004	109.88	10.24	67.40	147.00
**ADG, g/d**	4436	976.60	145.32	351.00	1492.00
**Ultrasound backfat depth, mm**	4810	13.73	3.17	5.20	28.70
**Ultrasound loin depth, mm**	4811	63.15	5.62	40.90	82.20
**Ultrasound IMF**	1807	1.26	0.83	0	6.60
**Feed conversion ratio**	708	2.64	0.30	1.55	4.06

### Heritabilities

Heritability estimates for performance traits with their standard errors are presented in Table 3 (diagonal elements). Univariate estimates of heritability for all traits were similar to the bivariate estimates. Moderate heritability was obtained for most of the performance traits with the estimates of 0.26, 0.24, 0.38, 0.30, 0.45, 0.38, 0.26, and 0.20 for BW, NURW, ENDW, ADG, UFD, ULD, UIMF, and FCR (Table 3). Weaning weight had a lower heritability of 0.07 in this study. These estimates were within the range (0.00–0.74) of the heritability previously reported for growth and performance traits [13], [14]. Several factors influence the heritability estimates, which may include the end-point adjustment such as age or weight adjustment, sampling, population size, effect of heterosis on crossbred populations and the completeness of pedigree [9], which may result in the various estimates among the different literature. The low-to-moderate heritability estimated from this study revealed that genomic technology can play an important role for improvement of these economically important traits.

**Table 3:** Estimates of genetic (below diagonal), phenotypic (above diagonal) correlations, heritabilities (diagonal) and their standard error of estimates among performance traits.

Traits^1^	BW	WNW	NURW	ENDW	ADG	UFD	ULD	UIMF	FCR
BW	***0.26±0.08***	**0.69±0.06** 2	**0.47±0.03**	**0.25±0.04**	**0.42±0.04**	−0.05±0.04	**0.10±0.04**	**0.13±0.05**	–
WNW	−0.00±0.74	*0.07±0.07*	**0.19±0.03**	**0.31±0.02**	**0.22±0.08**	**0.26±0.02**	**0.16±0.02**	**0.25±0.06**	–
NURW	−0.05±0.74	0.02±0.83	*0.24±0.16*	**0.50±0.03**	**0.52±0.03**	**0.26±0.02**	**0.18±0.04**	**0.47±0.06**	–
ENDW	**0.79±0.40**	**0.93±0.45**	−0.01±0.44	***0.38±0.08***	**0.80±0.01**	**0.31±0.02**	**0.41±0.02**	**0.36±0.04**	**0.33±0.04**
ADG	−0.68±0.70	0.06±0.67	−0.03±0.45	**0.87±0.04**	***0.30±0.08***	**0.27±0.02**	**0.31±0.02**	**0.32±0.03**	**0.31±0.04**
UFD	−0.36±0.40	0.03±0.83	−0.47±0.86	**0.28±0.13**	**0.26±0.12**	***0.45±0.07***	**0.08±0.02**	**0.34±0.04**	**0.28±0.04**
ULD	**0.75±0.36**	0.04±0.79	−0.48±0.49	**0.37±0.12**	**0.31±0.13**	−**0.33±0.14**	***0.38±0.07***	0.10±0.07	**0.21±0.05**
UIMF	0.33±0.39	0.00±0.72	**0.75±0.35**	**0.60±0.27**	**0.69±0.25**	**0.48±0.19**	−**0.47±0.20**	***0.26±0.06***	–
FCR	–	–	–	0.04±0.21	−0.19±0.20	**0.39±0.17**	0.05±0.21	–	***0.20±0.06***

1 **BW** = Birth weight (kg); **WNW** = Weaning weigh (kg); **NURW** = Nursery weight (kg); **ENDW** = End weight (kg); **ADG** = Average daily gain (g/d); **UFD** = Ultrasound backfat depth (mm); **ULD** = Ultrasound loin depth (mm); **UIMF** = Ultrasound IMF; **FCR** = Feed conversion ratio.

2 The significant correlations are bolded (P<0.05).

The estimated heritability for BW (0.26±0.08) in this study was higher than the estimates from many other work [13], [15], [16], [17], but lower than the report (0.36) by Roehe et al. [18], who used two-generations of outdoor reared piglets and suggested that direct heritability estimate was substantially larger under outdoor conditions. However, the estimate from the first generation of outdoor piglets also reported by Roehe et al. [19] was lower (0.20), which is close to our estimate from the crossbred population. Another difference is that Roehe et al. [18], [19] used a Bayesian method while we used an animal model. These results revealed that the breed, population structure and statistical method have an important effect on the genetic parameter estimate. The estimated heritability of WNW in this study (0.07±0.07) was in agreement with literature values [20]. The estimated maternal heritability for WNW was 0.10±0.03, which was similar to 0.17 reported by Cassady et al. [13]. Cassady et al. [13] estimated the heritability as 0.00 to 0.10 in two different genetic types. According to these studies, the maternal effect was a more important component of the genetic variation of weaning weight than the direct additive genetic effect. This may be due to effects of milk production, uterine capacity and nutrition to weaning [20]. The reports for genetic parameter estimates for NURW and ENDW are very limited, although they are important indicators to determine the production efficiency in the swine industry. The average ENDW was 110 (SD = 10) kg and this off test weight was not considered for heritability estimations in the literature. The NURW and ENDW heritability estimates were 0.24±0.16, and 0.38±0.18, respectively. The heritability for weight increased (0.07 to 0.38) from weaning to 160 days of age since the maternal genetic variance decreased as the pigs grew. This result is expected due to the separation of pigs from their dams. The common litter environment effect was fitted in the animal models for all performance traits except for UIMF and FCR.

ADG has been reported as a moderately heritable trait. The heritability estimate in this study is 0.30±0.08, which is in agreement with many other reports [14], [20]. However, van Wijk et al. [7] reported a lower heritability of 0.19, which may due to the different evaluation of ADG. van Wijk et al. [7] calculated the ADG based on the carcass weight and the assumption of the same birth weight of 1.36 kg for all animals, which could narrow down the sample variance and result in the low heritability estimation. Genetic parameters for ADG were widely studied and the reported estimates vary considerably, ranging from 0.03 to 0.49 [22]–[26]. The heritability of UFD in this study (0.45±0.07) was in good agreement with the previous report of 0.44–0.54 [7], [27]. Stewart and Schinckel [21] reviewed many papers and reported a weighted average heritability of 0.41 for backfat. The heritability estimate of ULD in the present study (0.38±0.07) was the same as the report (0.38) by Maignel et al. [28] who used the similar typical Canadian three-way cross population and sample size as our current study. However, the present estimate was slightly lower than the estimates of 0.47 and 0.48 reported by Stewart and Schinckel [21] and Ducos [29], respectively.

Marbling is one of the most important appearance factors used by consumers to perceive quality since they affect purchase decisions and satisfaction of consumption. The amount of marbling depends on implementation of different pig breeding and management techniques [23], which may be one of the reasons for the variation observed in the estimation of UIMF. The heritability of UIMF was moderate in the present study (0.26±0.06). UIMF has previously been reported to be a moderately heritable trait, ranging from 0.13 to 0.31, which was in agreement with the current result [23], [30], [31], [32]. The estimated heritability of FCR in this study was 0.20±0.06, which was lower than the average of 0.30 reviewed by Clutter [14], which may be due to using different statistical models, breeds and sample size.

Generally, meat quality traits had low-to-moderate (0.10±0.04 to 0.39±0.06) heritabilities while carcass composition traits had moderate-to-high (0.22±0.08 to 0.63±0.04) heritabilities. The details can be found from our previous report, which was conducted in the same population [2].

### Correlations among Traits

The phenotypic and genetic correlations and their standard errors are presented in Tables 3–7. Generally, almost all of the phenotypic correlations and some of the genetic correlations were significant (*P<0.05*). Although presented for completeness, phenotypic correlations will not be discussed because they are of little interpretive value.

**Table 4:** Estimates of phenotypic correlations and their standard error of estimates between carcass and performance traits.

Traits^1^	BW	WNW	NURW	ADG	UFD	ULD	UIMF	FCR
**HCW**	0.02±0.05	0.04±0.05	−0.03±0.04	**0.37±0.02** 2	**0.14±0.03**	**0.16±0.03**	**0.40±0.05**	**0.41±0.05**
**CCW**	0.02±0.05	0.04±0.05	−0.03±0.05	**0.37±0.02**	**0.13±0.03**	**0.16±0.03**	**0.40±0.05**	**0.41±0.05**
**FD**	−0.06±0.05	−0.07±0.04	**0.28±0.06**	**0.34±0.03**	**0.37±0.03**	**0.16±0.04**	−0.05±0.04	**0.32±0.05**
**LD**	0.07±0.04	−**0.09±0.04**	0.06±0.06	**0.32±0.03**	−**0.06±0.03**	**0.15±0.03**	0.03±0.04	−0.09±0.06
**CLEN**	0.00±0.05	0.01±0.05	**0.19±0.08**	**0.36±0.05**	**0.09±0.04**	**0.15±0.07**	0.04±0.05	−0.09±0.07
**LEA**	−0.01±0.05	−0.02±0.04	−0.00±0.04	**0.07±0.03**	−**0.10±0.03**	**0.12±0.03**	**0.32±0.06**	**0.27±0.07**
**TEXS**	**0.18±0.07**	−0.05±0.04	**0.41±0.08**	**0.32±0.03**	**0.17±0.05**	**0.19±0.05**	0.04±0.04	0.04±0.05
**CMAR**	0.05±0.05	−0.03±0.06	**0.36±0.09**	**0.33±0.03**	**0.15±0.05**	**0.17±0.05**	0.00±0.04	**0.12±0.05**
**USW**	0.02±0.05	0.04±0.05	**0.19±0.08**	**0.39±0.04**	**0.19±0.05**	**0.26±0.06**	0.04±0.04	0.09±0.07
**UHAM**	0.10±0.06	0.03±0.05	**0.37±0.09**	**0.34±0.05**	**0.26±0.07**	**0.31±0.07**	0.05±0.04	0.02±0.07
**ULOIN**	0.01±0.05	−0.01±0.05	**0.27±0.09**	**0.35±0.05**	**0.22±0.06**	**0.28±0.07**	0.02±0.04	0.14±0.08
**USH**	**0.12±0.06**	**0.09±0.04**	**0.36±0.10**	**0.36±0.05**	**0.24±0.08**	**0.29±0.07**	0.01±0.04	0.09±0.07
**UBEL**	0.07±0.06	−0.02±0.05	**0.37±0.10**	**0.35±0.05**	**0.33±0.07**	**0.30±0.07**	0.03±0.04	**0.18±0.07**
**THAM**	**0.17±0.05**	**0.17±0.04**	**0.35±0.10**	**0.35±0.05**	**0.23±0.08**	**0.28±0.08**	0.00±0.04	0.02±0.09
**TLOIN**	0.03±0.06	−0.01±0.05	**0.33±0.10**	**0.34±0.05**	**0.19±0.08**	**0.28±0.08**	0.04±0.04	0.03±0.10
**TBEL**	**0.24±0.05**	**0.14±0.04**	**0.53±0.04**	**0.29±0.04**	**0.33±0.07**	**0.32±0.07**	**0.18±0.04**	**0.26±0.08**
**PICN**	**0.35±0.06**	**0.12±0.05**	**0.42±0.06**	**0.28±0.04**	**0.33±0.07**	**0.29±0.07**	**0.11±0.04**	0.06±0.07
**BUTT**	**0.39±0.07**	**0.11±0.04**	**0.43±0.06**	**0.31±0.05**	**0.30±0.07**	**0.28±0.07**	**0.11±0.04**	−0.02±0.06
**RIBS**	**0.54±0.09**	**0.16±0.07**	**0.40±0.09**	**0.32±0.05**	**0.32±0.07**	**0.29±0.07**	**0.22±0.07**	0.06±0.07

1 **BW** = Birth weight (kg); **WNW** = Weaning weigh (kg); **NURW** = Nursery weight (kg); **ENDW** = End weight (kg); **ADG** = Average daily gain (g/d); **UFD** = Ultrasound backfat depth (mm); **ULD** = Ultrasound loin depth (mm); **UIMF** = Ultrasound IMF; **FCR** = Feed conversion ratio; **HCW = **Hot carcass weight (kg); **CCW = **Cold carcass weight (kg); **FD = **Backfat depth (mm); **LD = **Loin depth (mm); **CLEN = **Carcass length (cm); **LEA =** *Longissimus dorsi* muscle area (cm^2^); **TEXS = **Texture score; **CMAR = **Carcass marbling score; **USW = **Untrimmed side weight (kg); **UHAM = **Untrimmed ham weight (kg); **ULOIN = **Untrimmed loin weight (kg); **USH = **Untrimmed shoulder weight (kg); **UBEL = **Untrimmed belly weight (kg); **THAM = **Trimmed ham weight (kg); **TLOIN = **Trimmed loin weight (kg); **TBEL = **Trimmed belly weight (kg); **PICN = **Trimmed picnic shoulder weight (kg); **BUTT = **Butt shoulder weight (kg); **RIBS = **Ribs weight (kg).

2 The significant correlations are bolded (*P<0.05*).

**Table 5:** Estimates of genetic correlations and their standard error of estimates between carcass and performance traits.

Traits^1^	BW	WNW	NURW	ADG	UFD	ULD	UIMF	FCR
**HCW**	0.19±0.84	−0.06±0.13	−0.94±0.89	**0.75±0.28** 2	0.11±0.34	**0.20±0.10**	0.47±0.41	0.15±0.28
**CCW**	0.40±0.74	−0.07±0.12	−0.93±0.89	**0.78±0.27**	0.05±0.33	0.39±0.30	0.54±0.40	0.15±0.27
**FD**	−**0.69±0.30**	−0.85±0.68	−0.18±0.40	0.01±0.14	**0.53±0.12**	0.10±0.13	−0.22±0.28	0.20±0.20
**LD**	0.32±0.26	**0.39±0.14**	**0.69±0.27**	−0.10±0.13	−0.02±0.12	**0.39±0.12**	0.16±0.24	0.30±0.20
**CLEN**	0.18±0.59	−0.07±0.13	−0.18±0.33	**0.44±0.14**	0.05±0.14	0.10±0.13	0.15±0.23	−0.21±0.18
**LEA**	−0.25±0.73	−0.08±0.14	0.80±0.71	0.10±0.27	0.12±0.30	0.47±0.27	0.57±0.38	0.33±0.24
**TEXS**	−0.34±0.48	0.11±0.26	−0.29±0.42	−0.36±0.20	0.09±0.20	−0.24±0.20	−0.64±0.48	−0.03±0.33
**CMAR**	−0.38±0.34	−0.09±0.12	−0.32±0.31	0.05±0.15	−0.16±0.14	−0.09±0.14	**0.59±0.28**	0.38±0.23
**USW**	−0.19±0.26	−0.08±0.13	−0.10±0.33	**0.43±0.14**	0.09±0.14	**0.26±0.13**	0.24±0.24	0.18±0.17
**UHAM**	−0.18±0.26	−0.06±0.13	0.01±0.31	**0.34±0.14**	0.11±0.14	**0.29±0.13**	0.30±0.24	0.15±0.18
**ULOIN**	−0.25±0.19	−0.11±0.11	−0.01±0.26	0.13±0.13	0.14±0.13	**0.26±0.13**	0.17±0.19	0.34±0.18
**USH**	0.01±0.25	0.16±0.12	0.01±0.27	**0.35±0.14**	−0.09±0.14	0.17±0.13	−0.06±0.22	0.17±0.18
**UBEL**	−0.25±0.24	−0.17±0.12	0.10±0.28	**0.48±0.14**	**0.29±0.13**	0.21±0.13	0.18±0.23	0.21±0.18
**THAM**	0.22±0.18	**0.17±0.08**	0.12±0.21	**0.25±0.12**	−0.04±0.11	0.13±0.10	0.05±0.15	0.09±0.20
**TLOIN**	−0.24±0.22	−0.12±0.12	−0.01±0.27	0.18±0.16	0.06±0.15	0.14±0.14	0.22±0.21	**0.44±0.26**
**TBEL**	0.16±0.22	**0.19±0.05**	**0.91±0.11**	**0.77±0.07**	**0.29±0.13**	0.19±0.13	**0.75±0.22**	0.32±0.20
**PICN**	0.40±0.25	**0.27±0.13**	**0.94±0.13**	**0.79±0.08**	**0.29±0.14**	**0.23±0.11**	**0.77±0.25**	0.17±0.19
**BUTT**	0.50±0.30	0.26±0.16	**0.94±0.17**	**0.74±0.10**	−0.15±0.16	**0.34±0.13**	**0.82±0.22**	0.07±0.18
**RIBS**	0.45±0.47	0.29±0.22	**0.94±0.32**	**0.73±0.12**	−0.22±0.20	0.23±0.17	0.62±0.41	0.00±0.21

1 See Table 4 for trait abbreviation definitions.

2 The significant correlations are bolded (*P<0.05*).

**Table 6:** Estimates of phenotypic correlations and their standard error of estimates between meat quality and performance traits.

Traits^1^	BW	WNW	NURW	ADG	UFD	ULD	UIMF	FCR
**WLW**	**0.48±0.08** 2	**0.11±0.04**	**0.36±0.08**	**0.33±0.05**	**0.32±0.07**	**0.28±0.06**	**0.06±0.03**	0.09±0.07
**REAW**	**0.53±0.09**	**0.26±0.04**	**0.41±0.09**	**0.33±0.05**	**0.21±0.08**	**0.24±0.07**	−**0.16±0.04**	−0.10±0.06
**BFW**	**0.40±0.11**	**0.12±0.05**	**0.41±0.08**	**0.34±0.05**	0.31±0.54	**0.26±0.08**	**0.19±0.04**	**0.38±0.06**
**RTW**	**0.51±0.10**	**0.24±0.04**	**0.42±0.09**	**0.34±0.05**	**0.20±0.09**	**0.27±0.08**	0.00±0.04	−**0.19±0.06**
**BOW**	**0.50±0.10**	**0.18±0.04**	**0.41±0.09**	**0.33±0.05**	**0.26±0.08**	**0.27±0.08**	−0.07±0.04	**−0.18±0.06**
**CL**	0.04±0.04	−0.07±0.04	0.15±0.09	**0.33±0.03**	**−0.06±0.03**	**0.12±0.04**	−0.06±0.04	−0.01±0.06
**REAL**	−0.08±0.05	**−0.14±0.04**	**0.23±0.09**	**0.32±0.03**	**0.12±0.04**	0.06±0.05	**0.21±0.04**	0.07±0.06
**REAA**	**0.15±0.05**	**0.14±0.04**	**0.18±0.04**	0.02±0.03	**0.11±0.03**	**−0.06±0.03**	**0.22±0.05**	**0.21±0.08**
**REAB**	**0.13±0.05**	−0.03±0.04	**0.43±0.08**	**0.33±0.03**	**0.19±0.04**	0.11±0.06	**0.29±0.04**	**0.14±0.06**
**SHF**	−0.06±0.04	−0.03±0.04	−0.02±0.05	**0.30±0.04**	**−0.11±0.03**	−0.02±0.03	**−0.15±0.04**	−0.06±0.06
**LOINL**	**0.16±0.05**	0.00±0.04	**0.26±0.08**	**0.33±0.03**	**0.15±0.03**	**0.13±0.04**	−0.03±0.04	**0.12±0.06**
**LOINA**	**0.15±0.05**	0.03±0.04	**0.37±0.08**	**0.33±0.03**	**0.11±0.04**	**0.13±0.05**	−0.06±0.04	0.06±0.06
**LOINB**	**0.18±0.04**	0.03±0.04	**0.36±0.08**	**0.33±0.03**	**0.18±0.04**	**0.17±0.05**	−0.03±0.04	**0.14±0.06**
**PHU**	−0.03±0.04	−0.01±0.04	0.01±0.04	0.03±0.03	−0.02±0.03	−0.04±0.03	0.05±0.04	−0.01±0.06
**HGML**	**0.15±0.04**	0.01±0.04	**0.25±0.09**	**0.32±0.03**	0.00±0.04	0.07±0.04	0.04±0.04	0.03±0.06
**HGMA**	**0.15±0.05**	0.01±0.04	**0.30±0.10**	**0.32±0.03**	**0.11±0.05**	**0.13±0.05**	−0.06±0.04	0.02±0.06
**HGMB**	**0.10±0.05**	0.03±0.04	**0.41±0.08**	**0.33±0.03**	**0.10±0.04**	**0.15±0.05**	0.02±0.04	0.00±0.06
**HQFL**	0.04±0.05	−0.02±0.04	**0.25±0.07**	**0.33±0.03**	**0.06±0.03**	**0.08±0.04**	−0.03±0.04	0.06±0.06
**HQFA**	0.04±0.04	0.02±0.04	**0.34±0.08**	**0.33±0.03**	**0.10±0.04**	0.09±0.05	0.05±0.04	−0.08±0.05
**HQFB**	0.08±0.05	0.00±0.04	**0.42±0.06**	**0.33±0.03**	**0.12±0.04**	**0.13±0.05**	0.06±0.04	−0.00±0.06
**HILL**	**0.11±0.04**	0.01±0.04	**0.18±0.08**	**0.33±0.03**	**0.13±0.03**	**0.10±0.04**	0.03±0.04	0.03±0.06
**HILA**	0.07±0.04	0.03±0.04	**0.36±0.08**	**0.33±0.03**	0.05±0.04	**0.11±0.05**	0.01±0.04	0.04±0.06
**HILB**	**0.11±0.05**	−0.01±0.04	**0.30±0.09**	**0.32±0.03**	**0.14±0.04**	**0.13±0.05**	0.03±0.04	0.04±0.06
**DL**	**0.14±0.04**	0.02±0.04	−0.07±0.04	−0.02±0.03	**−0.06±0.03**	0.01±0.03	0.02±0.05	0.11±0.08

1 **BW** = Birth weight (kg); **WNW** = Weaning weigh (kg); **NURW** = Nursery weight (kg); **ADG** = Average daily gain (g/d); **UFD** = Ultrasound backfat depth (mm); **ULD** = Ultrasound loin depth (mm); **UIMF** = Ultrasound IMF; **FCR** = Feed conversion ratio; **WLW** = Whole loin weight (kg); **REAW** = Rib eye weight (kg); **BFW** = Backfat thickness weight (kg); **RTW** = Rib trim weight (kg); **BOW** = Bone/Neural weight (kg); **CL** = Cooking loss (%); **REAL** = Minolta L* rib eye area; **REAA** = Minolta a* rib eye area; **REAB** = Minolta b* rib eye area; **SHF** = Shear force (newton); **LOINL** = Minolta L* loin; **LOINA** = Minolta a* loin; **LOINB** = Minolta b* loin; **PHU** = pH ultimate; **HGML** = Minolta L* ham *gluteus medius*; **HGMA** = Minolta a* ham *gluteus medius*; **HGMB** = Minolta b* ham *gluteus medius*; **HQFL** = Minolta L* ham *quadriceps femoris*; **HQFA** = Minolta a* ham *quadriceps femoris*; **HQFB** = Minolta b* ham *quadriceps femoris*; **HILL** = Minolta L* ham *iliopsoas*; **HILA** = Minolta a* ham *iliopsoas*; **HILB** = Minolta b* ham *iliopsoas*; **DL** = Drip loss (%).

2 The significant correlations are bolded (*P<0.05*).

**Table 7:** Estimates of genetic correlations and their standard error of estimates between meat quality and performance traits.

Traits^1^	BW	WNW	NURW	ADG	UFD	ULD	UIMF	FCR
**WLW**	0.41±0.25	**0.20±0.07** 2	**0.80±0.18**	**0.52±0.13**	0.19±0.15	**0.42±0.13**	0.21±0.25	−0.05±0.24
**REAW**	0.43±0.31	**0.33±0.12**	−0.24±0.29	0.17±0.16	**−0.75±0.11**	**0.66±0.10**	−0.39±0.25	0.13±0.23
**BFW**	−0.33±0.27	0.08±0.14	**0.67±0.31**	−0.15±0.16	**0.89±0.05**	−0.17±0.14	0.36±0.23	0.24±0.19
**RTW**	−0.02±0.28	**0.34±0.14**	−0.09±0.40	**0.32±0.16**	**−0.66±0.11**	0.24±0.15	0.02±0.28	−0.28±0.22
**BOW**	0.09±0.34	**0.46±0.17**	−0.66±0.49	**0.43±0.19**	**−0.45±0.17**	**−0.36±0.18**	0.28±0.38	−0.33±0.26
**CL**	0.05±0.31	−0.02±0.18	**−0.51±0.24**	0.25±0.15	**−0.41±0.13**	0.11±0.15	**−0.67±0.26**	0.08±0.25
**REAL**	−0.11±0.25	−0.14±0.14	−0.10±0.22	0.02±0.14	**0.24±0.12**	0.04±0.13	**0.63±0.22**	0.29±0.20
**REAA**	0.63±0.44	0.22±0.13	−0.22±0.73	−0.14±0.18	0.05±0.17	−0.07±0.17	0.36±0.27	−0.07±0.22
**REAB**	0.36±0.34	0.03±0.15	0.23±0.23	−0.01±0.14	**0.24±0.12**	−0.14±0.12	**0.77±0.18**	0.18±0.22
**SHF**	−0.14±0.26	−0.04±0.13	−0.17±0.20	0.10±0.14	−0.14±0.12	−0.15±0.12	−0.30±0.23	−0.09±0.23
**LOINL**	**0.76±0.37**	−0.22±0.15	−0.06±0.41	0.08±0.14	0.12±0.13	0.20±0.13	−0.43±0.26	**0.43±0.19**
**LOINA**	0.50±0.34	−0.02±0.15	0.29±0.25	−0.00±0.14	−0.12±0.12	−0.12±0.12	−0.35±0.25	−0.18±0.21
**LOINB**	**0.86±0.43**	−0.17±0.17	0.41±0.26	0.11±0.16	0.05±0.14	0.11±0.15	−0.33±0.28	0.32±0.24
**PHU**	0.11±0.71	0.06±0.14	0.40±0.90	0.19±0.24	−0.43±0.25	**−0.49±0.24**	**0.73±0.37**	−0.30±0.30
**HGML**	**0.80±0.31**	−0.12±0.17	**−0.69±0.35**	0.00±0.16	−0.25±0.14	−0.13±0.14	0.42±0.29	0.30±0.23
**HGMA**	0.44±0.32	−0.00±0.15	−0.40±0.26	−0.20±0.13	0.05±0.12	−0.10±0.12	−0.23±0.26	−0.29±0.19
**HGMB**	0.79±0.49	−0.24±0.24	−0.11±0.65	−0.13±0.20	−0.18±0.19	−0.15±0.18	**0.73±0.36**	0.22±0.29
**HQFL**	0.02±0.33	−0.18±0.18	0.06±0.39	−0.07±0.16	−0.05±0.14	0.19±0.14	−0.24±0.31	0.15±0.23
**HQFA**	−0.40±0.30	−0.10±0.16	0.53±0.46	0.03±0.15	0.15±0.13	−0.09±0.13	0.14±0.29	−0.25±0.21
**HQFB**	−0.24±0.40	−0.30±0.24	−0.19±0.23	0.03±0.19	0.15±0.17	0.12±0.18	0.45±0.39	−0.03±0.28
**HILL**	0.41±0.28	−0.07±0.14	−0.01±0.25	0.22±0.14	**0.34±0.13**	0.10±0.13	0.28±0.24	0.20±0.22
**HILA**	0.37±0.29	0.28±0.17	0.21±0.35	−0.15±0.16	−0.01±0.14	0.01±0.14	−0.19±0.30	−0.29±0.22
**HILB**	0.45±0.30	−0.06±0.16	−0.11±0.26	0.16±0.15	**0.33±0.14**	0.08±0.14	0.34±0.26	−0.08±0.24
**DL**	**0.93±0.42**	0.07±0.13	−0.92±0.91	0.07±0.22	−0.15±0.21	−0.09±0.21	−0.25±0.34	0.13±0.27

1 See Table 6 for trait abbreviation definitions.

2 The significant correlations are bolded (*P<0.05*).

### Correlations among Growth and Performance Traits

The phenotypic and genetic correlations among growth and performance traits are presented in Table 3. Almost all of the phenotypic correlations among performance traits were significant (*P<0.05*). Genetic correlations indicated that selection for increased growth rate could increase ULD (0.31±0.13), UIMF (0.69±0.25), and UFD (0.26±0.12). Growth is in general lowly and negatively correlated with backfat thickness but favourably correlated with marbling and loin depth. The ADG and FD are the most important traits of performance testing, and the genetic correlation between them (0.01±0.14) is in the range of estimates (−0.26 to 0.55) reviewed by Clutter [14]. The wide range of genetic correlations between ADG and UFD reported by Clutter [14] may be due to the method of measurement, technician effect, breed differences, and sampling errors [33]. These results suggested that breeding programs aimed at improving intramuscular fat should expect improvement (higher marbling) through the selection for growth. Suzuki et al. [34] reported a low genetic correlation of 0.06 between UIMF and ADG that is lower than this study, which may be due to using the smaller samples size and purebred Duroc in their study. We highlight that the genetic correlation between ADG and ULD is a new contribution to our knowledge.

Birth weight had strong genetic correlations with ENDW (0.79±0.40) and ULD (0.75±0.36). Generally, genetic correlations of ENDW with performance traits were significant (*P<0.05*) except for the correlations with NURW and FCR. ENDW had high genetic correlations with BW (0.79±0.40), WNW (0.93±0.45), ADG (0.87±0.04), and UIMF (0.60±0.27). None of these genetic correlations were previously reported and it seems that selection for BW, WNW, and ADG will lead to increased ENDW and UIMF. Low-to-moderate correlations were found between ENDW with UFD (0.28±0.13), and ULD (0.37±0.12). Genetic correlations of ENDW with these traits were not reported in the literature. UFD had moderate genetic correlation with ULD (−0.33±0.14). This result was similar to the average value of −0.35 reported by Clutter and Brascamp [27] and −0.45 by Newcom et al. [35].

In addition, UIMF was moderately to highly correlated with NURW (0.75±0.35), ENDW (0.60±0.27), ADG (0.69±0.25), UFD (0.48±0.19), and ULD (−0.47±0.20). However, no genetic correlations were found for NURW and ENDW (−0.01±0.44) but these results confirmed that selection based on NURW would increase UIMF. These results also imply that increased backfat and decreased loin depth may be expected when selection is directed toward increased marbling. FCR was also moderately correlated with UFD (0.39±0.17), indicating that selection for lower FCR may result in greater backfat depth. Although the genetic correlation between ADG and FCR was not significant in the present study, a moderate to high and negative genetic correlation was reported by Clutter [14]. This difference may result from differences in sample size, breeds, and the feeding type. The nature of this discrepancy was not investigated further within the present study, but it warrants further examination.

#### Correlations between Performance and Carcass Traits

The phenotypic and genetic correlations between performance and carcass traits are presented in Tables 4–5. Almost all of the phenotypic correlations between performance and carcass traits were significant (*P<0.05*). Although, pork quality importance is increasing, pig breeders are only paid for carcass yield. Results of genetic correlations indicated that selection for BW would reduce the amount of backfat depth (−0.69±0.30), which was different from the report by Fix et al. [36] who demonstrated no significant (*P>0.05*) genetic correlation between BW and FD. The differences may be due to different statistical models.

However, selection for WNW and NURW would increase loin depth because of their moderate to high genetic correlations (0.39±0.14 and 0.69±0.27), respectively. To our knowledge, these estimates in the present study are a new contribution to the literature. Weaning weight had low genetic correlations with THAM, TBEL, and PICN (0.17±0.08, 0.19±0.05, and 0.27±0.13, respectively). Nursery weight was highly correlated with subprimal cuts including TBEL (0.91±0.11), PICN (0.94±0.13), BUTT (0.94±0.17), and RIBS (0.94±0.32). This implies that selection for high nursery weight will also lead to increased belly, picnic shoulder, and butt muscle yield. This study also indicates that the NURW should be recorded in pig breeding programs as an indicator trait for subprimal cuts selection. However, no genetic correlations for WNW and NURW with carcass traits were found in the literature.

Average daily gain is one of the most important traits of selection in the pig breeding programs. Based on the estimates of this study, genetic correlations between ADG and carcass yield were moderate to high, and selection on ADG would have favorable effects on carcass yield. In general, growth is moderately to highly correlated (averaging 0.47) with primal and subprimal cut weights. These results indicated that selection for higher growth could have an increasing effect on the most valuable primal and subprimal weights. However, our results were not in agreement with van Wijk et al. [7] who reported adverse effects (on an average of −0.29) of growth on some primal and subprimal weights. The discrepancy might be due to the different genetic background, less pedigree information, and smaller sample size in their study. In addition, growth is highly correlated to HCW (0.75±0.28) and CCW (0.78±0.27), which were not reported previously.

This study revealed that ultrasound measurements of backfat thickness, marbling score, and loin depth have moderate to strong genetic correlations with their corresponding measurements of carcass merit. The weakest genetic correlation (0.39±0.12) between ultrasound measures and their corresponding carcass measurements was observed between ULD and LD. This may be due to the difficulty of ultrasonic measurement of loin depth compared to backfat depth and marbling. Ultrasound backfat depth was correlated with UBEL (0.29±0.13), TBEL (0.29±0.13), and PICN (0.29±0.14). Again, to our knowledge, these estimates are new and imply that selection against ultrasound backfat depth would not necessarily reduce belly and picnic shoulder weights.

Low genetic correlations were estimated for ULD with HCW (0.20±0.10), USW (0.26±0.13), UHAM (0.29±0.13), ULOIN (0.26±0.13), PICN (0.23±0.11), and BUTT (0.34±0.13). These new results indicated that selection for high ultrasound loin depth may result in higher carcass, primal and subprimal yield including ham, loin, picnic shoulder and butt weight. High genetic correlations were also found between UIMF with TBEL (0.75±0.22), PICN (0.77±0.25), and BUTT (0.82±0.22). These imply that selection for high UIMF results in high trimmed belly, picnic shoulder and butt weight. This may be due to similar pattern of intramuscular fat deposition in these subprimal cuts. Feed conversion ratio was only correlated with TLOIN, indicating that selection for low FCR has no significant effect on carcass traits except of TLOIN (0.44±0.26). However, these results need to be further confirmed in a larger sample with FCR records.

#### Correlations between Performance and Meat Quality Traits

The phenotypic and genetic correlations between performance and meat quality traits are presented in Tables 6–7. Almost all of the phenotypic correlations between performance and meat quality traits were significant (*P<0.05*). However, a few significant (*P<0.05*) genetic correlations were found that can explain the hypothesis of negative effect of selection for performance traits on pork quality.

Several novel aspects were derived from this study in terms of the genetic correlation of birth weight, weaning weight, and nursery weight with pork quality. High genetic correlations were observed between BW with LOINL (0.76±0.37), LOINB (0.86±0.43), HGML (0.80±0.31), and DL (0.93±0.42). These results imply that selection for birth weight may increase drip loss, which could result in lighter color of loin *longissimus dorsi*, and ham *gluteus medius* muscles. No genetic correlations between BW and meat quality traits were found in the published literature. However, selection for high WNW does not affect pork quality but may increase the REAW (0.20±0.07), RTW (0.34±0.14), and BOW (0.46±0.17). These results indicate that selection for WNW will have no negative effects on pork quality. Moderate to high genetic antagonism was observed between NURW with CL (−0.51±0.24), and HGML (−0.69±0.35), which were also novel in this study. These results indicate that selection for high nursery weight will result in low cooking loss and lighter color of ham *gluteus medius.* However, NURW had low-to-moderate and favorable genetic correlations with other pork quality traits, indicating that selection for NURW does not have adverse effects on pork quality according to our study. The high genetic correlation found between NURW and BFW, indicating that selection for NURW will increase the backfat weight of rib eye area muscle.

Average daily gain, which is one of the main selection criteria, had no genetic correlations with all of the pork quality traits except for BOW and RTW, indicating that deterioration of pork quality was not occurring through selection for increasing ADG in these two populations. This is different to the report by van Wijk et al. [7] who showed unfavorable strong genetic correlations between growth rate and pork quality traits. However, De Vries et al. [5] and Hermesch et al. [37] reported no genetic correlation between growth and pork quality traits, which are similar to our results. In addition, ADG was correlated with RTW (0.32±0.16), and BOW (0.43±0.19) of rib eye area, which are also novel in this study. Ultrasound backfat depth was negatively correlated to REAW (−0.75±0.11), RTW (−0.66±0.11), BOW (−0.45±0.17), and CL (−0.41±0.13) but positively correlated to BFW (0.89±0.05). Low to moderate genetic correlations were also found between UFD with REAL (0.24±0.12), REAB (0.24±0.12), HILL (0.34±0.13), and HILB (0.33±0.14). These results indicate that selection for leaner carcass will not affect pork quality traits except for cooking loss and rib eye weight (Table 7). Most of the color traits were favorably correlated with UFD except for the lightness and yellowness of *iliopsoas* muscle of ham.

Unfavorable moderate genetic correlation was observed between ULD and PHU (−0.49±0.24). This was also different to the genetic correlation between carcass loin depth and pH observed by van Wijk et al. [7]. This might be due to the different method of loin depth measurement, genetic background, less pedigree information, and smaller sample size in their study. This indicates that single-trait selection on ultrasound loin depth may lead to undesirable lower pH pork. However, this result was similar to the genetic correlation between carcass loin depth and pH in this population [2]. Ultrasound loin depth was also correlated to REAW (0.66±0.10) and BOW (−0.36±0.18), which were not reported before. Unfavorable strong genetic correlations were obtained between UIMF with PHU (0.73±0.37), REAB (0.77±0.18), and HGMB (0.73±0.36). This indicates that selection on ultrasound intramuscular fat may lead to undesirable higher pH of meat with darker color. However, cooking loss was negatively correlated to UIMF (−0.67±0.26), indicating that increased UIMF will result in decreased cooking loss. Feed conversion ratio was only correlated with LOINL, indicating that selection for low FCR does not change pork quality except of lightness of loin (0.43±0.19). Genetic correlations between FCR and pork quality traits obtained in this study may be biased due to the small dataset available for FCR.

Some of the estimates herein are new contributions to the genetic correlations between performance traits with carcass and pork quality traits. Novel results from this study showed the nursery weight is an important trait and selection for NURW will have significant effects on carcass and pork quality traits through indirect selection. Novel genetic correlations in this study indicate that selection for birth weight, weaning weight, and growth may increase market weight, ultrasound loin depth, and ultrasound intramuscular fat. Favorable correlations were found between both weaning and nursery weight with loin depth. However, selection for nursery weight would lead to increased belly, picnic shoulder, and butt muscles yield, which were dissimilar with weaning weight. Birth weight has adverse effects on pork quality traits that may lead to undesirable higher drip loss pork with paler color but no adverse effect was found between weaning weight and pork quality. In addition, favorable genetic correlation was observed between nursery weight and pork quality, showing that selection for nursery weight may lead to increased carcass yield with no adverse effect on pork quality except for *gluteus medius* lightness. Therefore, selection for nursery weight can be valuable for increasing market weight and loin depth without adverse effects on pork quality traits.

Novel genetic correlations were obtained between growth and most valuable primal, subprimal, cold and hot carcass weight. These results indicate that selection for growth traits will increase carcass yield, which was dissimilar to selection for ultrasound loin depth. It was concluded that single-trait selection on ultrasound loin depth might lead to undesirable lower pH pork. However, no genetic effect was observed on water holding capacity. Therefore, selection for ADG can be valuable for increasing both carcass weight, primal and subprimal cuts weights. Selection for intramuscular fat may increase belly, picnic shoulder, butt weights, backfat thickness and reduce ultrasound loin depth and cooking loss with undesirable higher pH of meat with darker color. In addition, novel results show that selection for lower FCR may reduce backfat depth with no adverse consequences on pork quality traits except for paler loin, and selection for leaner carcass may affect pork quality traits including cooking loss and lightness of ham.

## Implications

Meat quality and carcass yield are important traits for the pork industry with consumers paying more attention to quality as well as value. Measurements of carcass and pork quality traits are difficult and expensive and can only be performed post-mortem. Genetic improvement of these traits is possible through indirect selection on performance traits, which requires knowledge of genetic parameters for these traits. However, the estimates of genetic correlations between carcass and pork quality with performance traits are limited despite its importance because the lack of measurement records of carcass and pork quality traits. In addition, segregation of the alleles from major loci is affecting the variation of pork quality traits in certain populations [38]. Therefore, understanding of genetic parameters for performance, pork quality, and carcass traits is essential for Canadian swine populations to implement efficient selection programs that emphasize product quality.

Genetic parameters obtained herein are valuable for the design of a breeding program emphasizing product quality in Canadian swine population. The low-to-moderate heritabilities of performance traits indicated that they could be improved using traditional breeding methods or genomic selection. Selection for high birth weight would have unfavorable consequences on pork quality traits including undesirable higher drip loss pork with paler color. It was concluded that selection for nursery weight would increase both quantity and quality traits. Furthermore, selection for ADG is also favorable for increasing carcass weight, primal and subprimal cuts weights with no adverse effects on pork quality. However, selection for intramuscular fat may affect pork quality traits but selection for FCR may reduce the lightness of loin. These results imply that selection for leaner carcass may affect cooking loss and lightness of ham. Although, these results indicated that deterioration of pork quality may have occurred over many generations through the selection for lower backfat thickness, and feed efficiency, but selection for growth had no adverse effects on pork quality traits. The genetic parameters identified here are valuable for understanding the biology of these traits making it possible to improve them simultaneously resulting in high quality product produced more efficiently and at lower cost.
